# Goldmann and modified Goldmann tonometry measuring intraocular pressure changes in eyes which underwent myopic laser in situ Keratomileusis and photorefractive keratectomy

**DOI:** 10.1186/s12886-022-02741-z

**Published:** 2022-12-20

**Authors:** Robert Edward T. Ang, Andrew Rixon, Khin Kilgore, Justin Schweitzer

**Affiliations:** 1 Department of Ophthalmology, Cardinal Santos Medical Center, City, Metro Manila, San Juan, Philippines; 2 Cornea and Refractive Surgery Service, Asian Eye Institute, City, Metro Manila, Makati, Philippines; 3 Glaucoma Service, Asian Eye Institute, City, Metro Manila, Makati, Philippines; 4 Veterans Administration, Memphis, TN U.S.A.; 5 Arizona Eye Consultants, Tucson, AZ U.S.A.; 6 Vance Thompson Vision, Sioux Falls, SD U.S.A.

**Keywords:** IOP, Tonometer, Goldmann, Glaucoma, GAT, CATS, LASIK, PRK, Cornea, Biomechanics

## Abstract

**Purpose:**

Compare intraocular pressure (IOP) measured by a standard Goldmann applanation tonometer prism (IOPg) and a modified correcting applanation tonometer surface Goldmann prism (IOPc) before and after laser in situ keratomileusis (LASIK) and photorefractive keratectomy (PRK).

**Methods:**

Goldmann tonometry was analyzed in a retrospective, cross-sectional study, using both GAT and modified-GAT prisms pre-operatively and at the 3 month post-operative appointment on 120 eyes (64 patients) who received LASIK (*n* = 58) or PRK (*n* = 62). Demographics, central corneal thickness (CCT), manifest refraction and corneal curvature (CC) data was collected at each visit as well as surgical parameters, including maximum ablation depth.

**Results:**

Mean paired IOP following LASIK decreased by − 3.28 ± 3.2 mmHg measured by IOPg and − 1.93 ± 3.3 mmHg by IOPc (*p* ≤ 0.0001). Mean paired IOP following PRK reduced by − 1.92 ± 3.6 mmHg measured by IOPg and − 1.06 ± 3.6 mmHg by IOPc (p ≤ 0.0001). Increased LASIK ablation depth and post-procedural change in CCT trended toward a statistically significant reduction in IOPg (*p* = 0.07,*p* = 0.12), but not IOPc (*p* = 0.18,*p* = 0.32). PRK ablation depth was not associated with a reduction in IOPg or IOPc.

**Discussion:**

The modified Goldmann (IOPc) prism measured less of an IOP reduction following LASIK and PRK compared to the standard (IOPg) prism, and the IOP reduction with both prisms was associated with the degree of myopic correction.

**What is already known and the residual query:**

Corneal refractive surgery generally demonstrates significant postoperative Goldmann IOP reductions. Presumably, this is due to corneal biomechanical changes for which a newer method of Goldmann IOP measurement may be able to compensate.

**What this study adds:**

A modified, corneal conforming Goldmann prism demonstrates significantly less IOP reduction following myopic LASIK and PRK compared to the standard flat Goldmann prism.

**How this study might affect research, practice or policy:**

A newer, modified Goldmann prism may help detect glaucoma and OHT at an earlier stage in patients which have undergone LASIK or PRK. The findings corroborate predicted corneal biomechanical changes following the most common corneal refractive procedures.

**Supplementary Information:**

The online version contains supplementary material available at 10.1186/s12886-022-02741-z.

## Synopsis

A corneal biomechanical correcting Goldmann prism measures significantly less IOP reduction following LASIK and PRK compared to a standard Goldmann prism. The IOP reduction tends to be correlated to procedural thinning of the cornea.

## Introduction

Intraocular pressure (IOP) measurement is critical to risk categorization and effective treatment of many disease processes of the eye. Furthermore, IOP is the most scrutinized indicator of progression in glaucomatous optic neuropathy (GON) and remains the clinically modifiable parameter in its treatment [1]. By convention, Goldmann Applanation Tonometry (GAT) has been the standard for IOP measurement [2]. Though a patient’s central corneal thickness (CCT), corneal curvature, elastic modulus, and tear film have demonstrated significant GAT IOP measurement errors [3–5]. Thin CCT has been associated with GON progression despite stable IOP to those with nominal CCTs [6–9]. Although CCT has been identified as an independent risk factor for GON, its association with inaccurate IOP measurement may be a contributing factor [10–12].

Corneal refractive surgeries (CRS), which include LASIK and PRK, are completed for myopia correction. Both procedures utilize excimer laser ablation, either on the stromal surface in PRK or under a partial thickness stromal flap in LASIK. PRK and LASIK have been shown to alter the corneal biomechanical properties [13–15]. Myopic correction using LASIK and PRK demonstrate significant postoperative GAT IOP reductions, presumably related to deeper central ablation depths [13–17]. The most accurate predictive parameter of post-refractive procedure IOP has been shown to be pre-refractive procedure IOP [13, 16, 17].

The correcting applanation tonometry surface prism is a modification of the flat-surfaced GAT prism; it uses a centrally concave and peripherally convex surface, which partially matches corneal curvature. The prism was designed to minimize the IOP measurement errors found with the GAT prism in a standard population [18]. The modified prism requires no Goldmann tonometer recalibration and uses the same GAT measurement protocol [19–23]. Clinical studies have demonstrated that the modified prism IOPc measurements are less sensitive to variations in CCT, corneal hysteresis (CH), and tear fil m[19–23]. Similar corneal conforming mechanisms of IOP measurement which reduce the effect of alterations in CCT following PRK have been demonstrated with dynamic contour tonometry (DCT ) [24]. The commercially available CATS prism used in this study should be differentiated from the convex Goldmann prism described in other studies [25]. The convex design was previously analyzed with normal corneal parameter variations (not LASIK) and found to develop a false minima in reducing corneal biomechanical IOP errors [18, 26].

The present study was completed to evaluate the differential effect before and after LASIK and PRK on Goldmann IOP measurement with the standard GAT IOPg and modified IOPc prisms. Study design complies with International Standards Organization (ISO) 8612:2009.

## Methods

The study is cross-sectional, retrospective design from a single outpatient center including all patients having undergone LASIK surgery or PRK. The study was completed in adherence to the tenets of Declaration of Helsinki and the Data Privacy Act of 2012. Ethical approval was obtained in advance of the study by the Ethics Review Committee, Saint Francis Cabrini- Asian Eye Institute (ERC#2021–004, February 22, 2021), informed consent was not required.

### Description of study population

Patients included those 18 years or older who had completed uneventful myopic correction with LASIK or PRK in one or both eyes. All patients had IOP measurements by Goldmann tonometry using both a standard GAT IOPg prism and a modified IOPc prism pre-operatively and at the 3 month post-operative visit. All LASIK and PRK procedures completed between May 1, 2020 and June 1, 2021 were analyzed and included IOP measured with both prisms. Only eyes that underwent myopic correction with a target refraction of emmetropia were included. For the purposes of ophthalmologic evaluation, only IOPg was considered. Chart review exclusion criteria encompassed: Previous ocular surgeries, glaucoma, ocular hypertension, glaucoma suspect, corneal pathologies, corneal scars, corneal edema and dystrophies. Patients with incomplete or untimely follow-up data were also excluded. A 60-eye sample size for both LASIK and PRK groups was estimated to provide sufficient power based upon prior study quantitative data and two group comparison power calculation with statistically significant probability of 0.05 [19–23].

### Protocol

Enrolled subjects received a complete ophthalmic exam pre-operatively and at the 3 months post-operative visit. Collected data on both visits included best and uncorrected visual acuity, slit lamp biomicroscopy, age, gender, manifest refraction, central corneal thickness (CCT), average corneal curvature, intraocular pressure by IOPg and IOPc, procedure (LASIK or PRK), and the maximum ablation depth. A manual phoropter was used to measure manifest refraction. Central corneal thickness and average corneal curvature were measured at both visits with the Pentacam (Oculus Optikgerate GmbH, Wetzlar, Germany, software version: 6.08r33). Goldmann IOP was measured with a calibrated Goldmann tonometer (model: AT900, Haag-Streit, Koeniz, Switzerland) utilizing both the modified prism (model: CATS-R, Tucson, Arizona) and standard GAT prism (model: AT900, Haag-Streit, Koeniz, Switzerland). Excimer laser maximum ablation depth was collected from the post procedural laser data (Teneo II, Bausch and Lomb, Germany). A 3 month follow up was chosen as patients demonstrate stable CCT and refraction as well as avoiding potential steroid response effects on the IOP. All data was collected in less than 3 weeks pre-operatively and at 3 months (±1.2 weeks) post-operatively. Modified IOPc and GAT IOPg measurements were completed by one of three trained clinicians. Measurement was preceded by instillation of topical proparacaine with fluorescent dye. Goldmann applanation tonometry was completed with the GAT prism followed by the modified prism with at least 3 min between measurements and without astigmatism axis averaging (astigmatism averaged 1.1 ± 0.9 diopters pre-operatively and 0.3 ± 0.3 post-operatively). All LASIK enrollees received a 120 μm flap with a femtosecond laser (Victus, Bausch and Lomb, Germany). Standard flap retraction with irrigation and drying followed by the prescribed excimer laser photoablation was performed (Teneo II, Bausch and Lomb, Germany) with immediate flap repositioning. All PRK enrollees received removal of the epithelium. This was completed either manually with topical alcohol application for 30 seconds with manual debridement, or with transepithelial PRK using the excimer laser (Teneo II, Bausch and Lomb, Germany). The prescribed ablation was performed and a bandage contact lens was applied.

### Endpoints

The primary endpoint was IOP measurement comparison between GAT IOPg and modified IOPc prisms before and after either a LASIK or PRK procedure. Secondary endpoints included IOP correlations between differential IOP (Mod.GAT-GAT) to both pre-operative, procedural, and post-operative data.

### Statistical methods

The Full Analysis Set (FAS) included all qualified eyes in the primary and secondary endpoint analysis. A database search was performed for all subjects completing refractive screening with both IOPc and IOPg measurements and uncomplicated LASIK or PRK surgery from May 1, 2020 to June 1, 2021. The subsets of LASIK and PRK subjects were analyzed separately. Data was anonymously transferred to the study record spreadsheet. (Microsoft Excel 2021, software version 16.45). Statistical analyses were performed with SPSS statistical software (SPSS 2018, version 20.0 software, IBM, Corp, Armonk, NY, USA). Continuous variables were used for descriptive statistics including mean, standard deviation, median, and range. The primary endpoints were analyzed using a homoscedastic or paired, 2-tailed t-test (α = 0.05). A linear regression Pearson bivariate correlation analysis examining the difference in paired pre-procedural minus post-procedural IOP with both IOPc and IOPg measurements to the measured and ablation predicted CCT changes. A multiple linear regression analysis was completed on the FAS using a general linear mixed effects (GLME) model examining Manifest Refraction Spherical Equivalent (MRSE), CC, CCT, IOP, ablation depth, age, and gender.

MRSE, CC, CCT, and IOP values were analyzed using homoscedastic or paired, 2-tailed t-test (α = 0.05) in a post hoc analysis to determine mean differences between groups. Bland-Altman analysis was used to evaluate the level of agreement between IOPc and IOPg measurements. Standard statistical significance was set to *p* ≤ 0.05. The clinical study was conducted within the ethical principles contained in Declaration of Helsinki, Code of Federal Regulations (CRF), Protection of Human Volunteers (21 CFR 50), Obligations of Clinical Investigators (21 CFR 812), and Institutional Review Boards (21 CFR 56).

## Results

One hundred and twenty unique eyes were examined before and after CRS treatment on 61 patients. There were 58 post-LASIK eyes with an average age of 30 ± 8 years and 62 post-PRK eyes with an average age of 30 ± 8 years meeting all the inclusion/exclusion criteria. There were 33 (57%) females enrolled in the LASIK cohort and the PRK cohort included 38 (61%) females. The mean, standard deviation, and range of all collected pre-operative data is compiled in Table 1. The pre-operative bivariate Pearson correlation data to the measured, paired IOP differences between IOPc and IOPg prisms is presented in Table 2. The pre-operative data sets were not significantly associated with the (IOPc-IOPg) differential IOP except MRSE, which was positively correlated. (*p* = 0.004, bivariate and *p* = 0.045 GLME).

**Table 1:** Pre-operative data in unique eyes of LASIK and PRK cohorts

Pre-Operative Data	LASIK +/−S.D. (Range)	PRK +/− S. D (Range)	Combined +/−S.D. (Range)
Number n=	58	62	120
Mean Age (yrs)	30+/−8 (50,18)	29.9+/−8 (52,19)	30+/− 8 (52,18)
Gender M/F	25/33	24/38	49/71
Mean MRSE (Diptrs)	(−)3.99+/− 1.58 (− 1,-7.6)	(−)5.1+/− 1.6 (− 1.4,-8.3)	(−)4.57+/− 1.68 (− 1,-8.25)
Mean CCT (Microns)	562+/− 25 (606,505)	538+/− 37 (616, 413)	550+/− 34 (616,413)
Mean CC (Dioptrs)	43.4+/− 1.2 (45.9,41.2)	43.5+/− 1.3 (47.1,41.0)	43.5+/−1.3 (47.1,41.0)
Mean IOPg (mmHg)	14.8+/−3.0 (20,7)	14.3+/− 2.6 (20,8)	14.5+/− 2.8 (20,7)
Mean IOPc (mmHg)	16.5+/− 2.8 (22,10)	16.1+/− 2.7 (22,10)	16.3+/− 2.7 (22,10)
Mean IOPc-IOPg (mmHg)	1.7+/−2.0 (8,-6)	1.8+/− 1.0 (4,0)	1.74+/− 1.54 (8,-6)

**Fig. 1:**
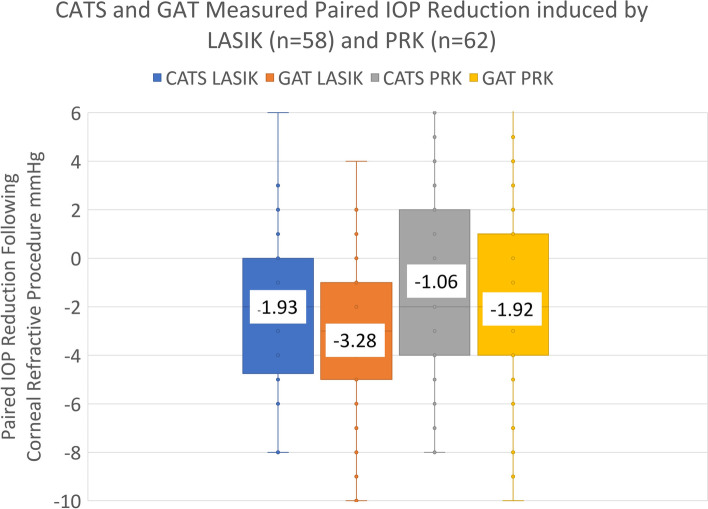
Box-whisker plot of IOP reduction following LASIK and PRK, measured by modified IOPc and standard IOPg prisms

**Table 2:** Pre-operative bivariate Pearson correlation data to the measured, paired IOP differences between IOPc and IOPg in combined LASIK and PRK patient pool (*n* = 120)

Pre-Operative Correlations	Pearson Corr. Coef. r=	Probability p=
CCT (Mod. GAT-GAT)	0.02	0.82
Age (Mod. GAT -GAT)	0.12	0.2
CC (Mod. GAT -GAT)	0.14	0.13
MRSE (Mod. GAT -GAT)	0.26	**0.004**
Bland-Altman (Mod. GAT -GAT)	(−)0.04	0.64

*IOPg and IOPc Reduction Analysis Following LASIK and PRK.* The paired IOP reduction following LASIK measured with IOPg and IOPc was −3.27 ± 3.01 mmHg and 1.93 ± 3.30 mmHg, respectively (Table 3). Following PRK, the paired IOP reduction measured with the GAT and Mod. GAT prisms was − 1.92 ± 3.60 mmHg and − 1.06 ± 3.58 mmHg, respectively. Box-whisker plots of the paired IOP reduction following both CRS procedures shows IOP was reduced post-operatively both prisms, but to a significantly lesser degree with IOPc (Fig. 1)

**Table 3:** IOPc and IOPg reduction following LASIK and PRK procedures including paired (post-operative – pre-operative) IOP measurement changes

Procedural Changes in IOP	LASIK (*n* = 58)	T-test between groups p=	PRK (*n* = 62)	T-test between groups p =
IOPg (GAT)				
Mean Pre-Op	14.8+/−3.0 (20,7)		14.3+/−2.6 (20,8)	
Mean Post-Op	11.5+/−2.0 (18,8)	< 0.0001	12.4+/−3.1 (19,6)	< 0.0001
IOPc (Mod. GAT)				
Mean Pre-Op	16.5+/−2.8 (22,10)		14.3+/−2.6 (20,8)	
Mean Post Op	12.8+/−2.1 (18,10)	< 0.0001	13.3+/−3.0 (19,6)	< 0.0001
Paired Procedural IOP Change				
IOPg (Post-Pre)	(−)3.27+/−3.2 (4,-10)		(−)1.92+/−3.6 (7,-10)	
IOPc (Post-Pre)	(−)1.93+/−3.3 (8,-8)	< 0.0001	(−)1.06+/− 3.6 (7,-8)	< 0.0001

**Fig. 2:**
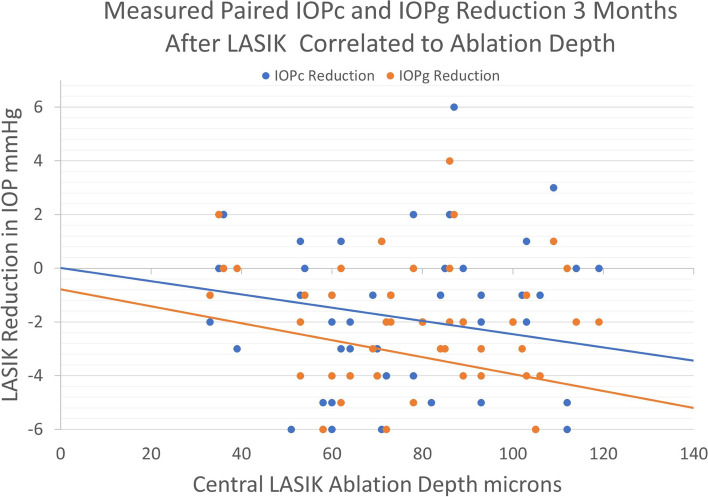
Measured modified correcting prism (IOPc) and standard GAT prism (IOPg) reduction following LASIK correlated to central ablation depth

*IOPc and IOPg Reduction with Ablation Depth and CCT Reduction.* Correlations to the measured parameters following LASIK and PRK were not statistically significant (Tables 4 and 5). It should be noted, however, that increased ablation depth and decreased CCT trended toward lower post-operative IOP measured with IOPc and IOPg (Fig. 2). The GAT reduction in IOP following LASIK was nearly significant following LASIK (−)0.24 mmHg/μm (*p* = 0.07)

## Conclusions

Goldmann IOP measurement is significantly reduced following both LASIK and PRK corneal refractive procedures for myopia. These results corroborate long-standing findings in myopic CRS IOP reduction.25 The modified correcting prism demonstrated significantly less IOP reduction following LASIK and PRK compared to the standard GAT prism. The IOP reduction following both LASIK and PRK is likely an error due to CCT thinning and corneal biomechanically-induced errors in Goldmann pressure measurement, although there was no direct intracameral IOP measurement for comparison [13]. A prior publication has demonstrated decreased CCT sensitivity to IOPc measurement when comparing IOPc and IOPg to intracameral pressure [22]. The IOP following myopic LASIK and PRK trended toward a greater reduction with increased ablation depth and measured CCT reduction. The IOPc measurement tended to be less effected by the ablation depth, but did not reach statistical significance. This correlation of IOP reduction to greater CRS myopic treatment has been demonstrated previously and is a postulated parameter in methods for post-CRS IOP correction [13, 27]. Although corneal biomechanics appear stable as demonstrated by MRSE and CCT stability at 3 months, there is the possibility of continued changes at 3 to 12 months following LASIK and PRK.

It is likely that these results indicate a global change in corneal rigidity with myopic LASIK and PRK. Corneal rigidity is a function of the cornea’s intrinsic elastic modulus which is multiplied by the scalar CCT. The interaction of CCT and corneal rigidity is depicted in Fig. 4, with a reduction of slope in Goldmann measured IOP to CCT. 3 Fig. 4 uses the Goldmann-CCT IOP model to demonstrate the probable differences seen in IOPc and IOPg measurements following LASIK. Both the modified correcting and standard GAT prisms were designed to measure the same Goldmann IOP in a nominal thickness cornea without corneal surgery [20]. The model describes a decreased slope with decreasing corneal rigidity due to a loss of structural resistance in the disrupted anterior lamellae following LASIK [3]. The IOPc is less affected by changes in CCT and likely changes in corneal rigidity, as well [19]. This model would predict both IOPc and IOPg measurements to be reduced following LASIK, but to a lesser degree with the modified correcting prism (IOPc). Likewise, the model would predict a similar observation but less dramatic results following PRK as less anterior stroma is disrupted. The observed findings support the model’s prediction in both cases.

**Table 4:** Post-operative correlations to parameters following LASIK. Bold approaches statistical significance

Post-Operative Cor. LASIK	Pearson Corr. Coef. r=	Probability p=
CCT (IOPc-IOPg)	0.07	0.61
Age (IOPc-IOPg)	0.14	0.2
Ablation Depth/IOPg Reduct	(−)0.24	**0.07**
Ablation Depth/IOPc Reduct	(−)0.18	0.18
CCT Change/IOPg Reduct	(−)0.21	0.12
CCT Change/IOPc Reduct	(−)0.13	0.32
Bland-Altman (IOPc-IOPg)	0.06	0.65

**Fig. 3:**
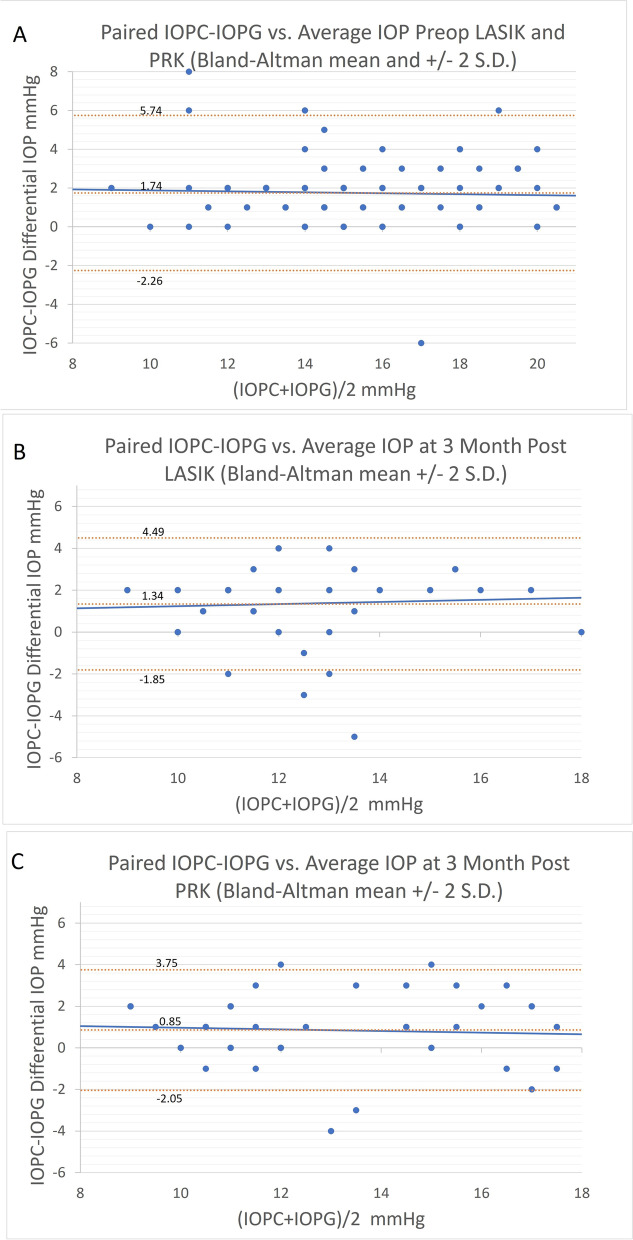
(A)Bland-Altman IOP measurement agreement plot in the combined pre-procedural LASIK and PRK study populations (with mean and ± 2 S.D. reference lines). (B)Bland-Altman IOP measurement agreement plot following LASIK (with mean and ± 2 S.D. reference lines).(C)Bland-Altman IOP measurement agreement plot following PRK (with mean and ± 2 S.D. reference lines)The general linear mixed effects (GLME) model indicated no significant correlations between the difference in IOPc and IOPg measurements to CCT, age, ablation depth, or CC. The GLME differential IOP correlation to MRSE was significant (*p* = 0.043) and GAT correlation to ablation depth again trended toward significance (*p* = 0.10).

**Table 5:** Post-operative correlations to parameters following PRK

Post-Operative Cor. PRK	Pearson Corr. Coef. r=	Probability p=
CCT (IOPc-IOPg)	(−)0.13	0.3
Age (IOPc-IOPg)	0.06	0.65
Ablation Depth/IOPg Reduct	(−)0.03	0.78
Ablation Depth/IOPc Reduct	(−)0.01	0.93
CCT Change/IOPg Reduct	(−)0.08	0.56
CCT Change/IOPc Reduct	(−)0.07	0.44
Bland-Altman (IOPc-IOPg)	(−)0.08	0.54

*IOPc and IOPg Measurement Agreement and Multi-variate analysis.*

Bland-Altman IOP measurement agreement analysis demonstrated a significant bias between the two measurements in the study population for both the pre- and post-LASIK and post-PRK groups (Fig. 3-A,B,C). There was no significant differential IOP correlation to the average IOP (Tables 2, 4 and 5)

**Fig. 4:**
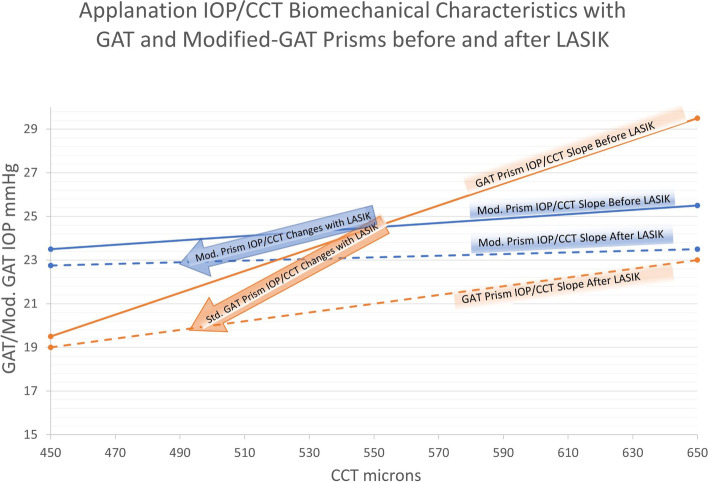
Goldmann IOP correlation to CCT in both modified correcting prism (IOPc) and standard GAT prism (IOPg) measurements before and after LASIK

A significant bias between IOPc and IOPg measurements was demonstrated in the study population before and after the CRS procedures. This finding was not seen in prior studies over broad ranging populations in age, CCT and MRSE [20]. Furthermore, the differential IOP between the two prisms did not significantly correlate to CCT as shown previously [19]. The comparatively narrow range of age and MRSE characteristics in the study population may have contributed to the differences in findings. Additionally, there was a significant positive correlation in pre-operative differential IOPc-IOPg to MRSE. Intuitively, the authors would have predicted this to be a negative correlation with thinner CCTs seen with a higher myopic MRSE. However, the presented data showed no significant correlation between preoperative MRSE and CCT. There may, however, be a significant correlation between intrinsic corneal rigidity and MRSE. A reduced elastic modulus (reduced rigidity) would decrease the differential IOPc and IOPg measurements. Unfortunately, the study includes no direct measurement of corneal rigidity except the IOPc-IOPg differential IOP to support this finding.

Future studies may include examining the difference in IOPc-IOPg measurement before and after LASIK with comparison to intracameral pressure in cadaver eyes. Goldmann IOP remains the standard of care for measuring IOP and the modified prism replacement to Goldmann may improve the fidelity of pressure measurement in surgically altered corneas.

## Supplementary Information

**Additional file 1.**

## Data Availability

The datasets used and/or analysed during the current study available from the corresponding author on reasonable request.

## Abbreviations

GAT: Goldmann applanation tonometer (prism)
IOPg: GAT Intraocular Pressure
IOPc: Modified prism GAT Intraocular Pressure
CATS: Correcting applanation tonometry surface (prism)
CCT: Central Corneal Thickness
FDA: Food and Drug Administration
ANSI: American National Standards Institute
ISO: International Standards Organization
CFR: Code of Federal Regulations
MRSE: Manifest Refraction Spherical Equivalent
CC: Corneal Curvature
IRB: Independent Review Board
